# From lifetime stressor exposure to daily stress experience: Associations with hair cortisol

**DOI:** 10.1016/j.cpnec.2026.100348

**Published:** 2026-04-08

**Authors:** Katharina Huthsteiner, Johannes B. Finke, Jari Planert, George M. Slavich, Tim Klucken, Tobias Stalder

**Affiliations:** a Department of Psychology, University of Siegen, Siegen, Germany; b Department of Psychiatry and Biobehavioral Sciences, University of California, Los Angeles, CA, USA

**Keywords:** Hair cortisol, DHEA, Chronic stress, Ecological momentary assessment, Early-life adversity

## Abstract

**Background:**

Although hair cortisol is an established index of long-term cumulative cortisol output, associations with chronic stress have been inconsistent, potentially due to heterogeneous and temporally misaligned stress assessments. Here, we integrate detailed lifetime stressor measures and ecological momentary assessments (EMA) to examine associations with hair cortisol and the cortisol/DHEA ratio.

**Methods:**

Two methodologically aligned studies were conducted. Study I (*N* = 79; cross-sectional) assessed self-reported cumulative lifetime stressor exposure using the Stress and Adversity Inventory (STRAIN), distinguishing early-life and adulthood stressor exposure. Study II (*N* = 86; two time points, three months apart) additionally assessed recent stress using the three-month STRAIN and repeated EMA. Hair hormone concentrations were quantified in proximal 3 cm segments via LC-MS/MS.

**Results:**

In Study I, neither early-life nor adulthood stressor exposure were associated with hair cortisol. In Study II, greater severity of adulthood stressor exposure was related to elevated hair cortisol concentrations, stable across time points and robust to covariate adjustment. Early-life and recent stress measures were not related to hair cortisol. Across both studies, the hair cortisol/DHEA ratio showed no consistent associations with any stress dimension.

**Conclusions:**

Our findings provide partial support for a positive association between self-reported adulthood stressor exposure and hair cortisol. However, the absence of consistent effects across both studies, despite substantial methodological overlap, echoes the heterogeneity reported in prior research. Potential reasons are discussed. Future research should address the complexity of individual stress exposure, incorporate hair cortisol into multimethod HPA axis assessments, and advance methodological standardization.

## Highlights

• Multiple stress dimensions and hair cortisol examined across two studies.
• Greater adulthood stressor exposure related to higher hair cortisol in Study II.
• No associations with early-life stressor exposure or recent three-month stress.
• Findings highlight the complexity of stress-hair cortisol associations.
• Multi-method stress hormone assessments in high-stress groups may advance the field.

## 1 Introduction

Chronic stress has been related to alterations in hypothalamic–pituitary–adrenal (HPA) functioning and ensuing adverse health effects [1], which makes the assessment of long-term HPA axis activity highly relevant for psychoneuroendocrine research. Hair cortisol has emerged as a promising biomarker to assess cumulative cortisol output over prolonged periods of time in that it provides a more convenient alternative to traditional sampling methods, such as blood, saliva, or urine [[2], [3], [4]]. Although hair cortisol analysis has demonstrated high reliability and validity as an indicator of long-term cortisol output [[5], [6], [7]], findings on its association with stressor exposure and subjectively perceived stress levels have been inconsistent.

In this context, prior research has found elevated hair cortisol in groups exposed to long-lasting stressful situations (e.g., athletes, shift workers, unemployment, chronic pain, natural disasters), particularly when the stressor is still ongoing (meta-analysis: [8]; review: [9]). By contrast, associations between hair cortisol and self-reported stress perception, as measured with brief questionnaires such as the Perceived Stress Scale (PSS) or the Trier Inventory for Chronic Stress (TICS), tend to be weak or absent [8,[10], [11], [12]]. This apparent discrepancy may partly be explained by methodological limitations of stress assessments [11,13]. In particular, self-report questionnaires are susceptible to several forms of bias including retrospection bias, social desirability, mood, and differences in awareness of affective state, all of which may weaken and potentially confound results [4]. Potential issues of retrospection bias may be particularly severe in hair cortisol research, as participants must accurately recall stress experiences over several months to correspond with the temporal window captured by hair cortisol measurements. These considerations highlight the need for more systematic, comprehensive, and temporally aligned assessment approaches in hair cortisol research [8]. One such approach involves using structured life stress interviews, which can assess different aspects and time spans of stressor exposure [14] and ecological momentary assessments (EMA), providing cumulative daily stress assessments less affected by retrospection and mood-congruent biases [15].

Consistent with this approach, some studies examining the effects of stress on hair cortisol have integrated assessments of cumulative stressor exposure across the lifespan. Elevated hair cortisol levels were found in individuals with recent exposure to a major life stressor, such as severe illness, death of a relative, or divorce [16]. Meta-analytic evidence further supports a positive association between various adverse experiences (e.g., natural disasters, maltreatment, trauma) and hair cortisol [17]. More recent research using the Stress and Adversity Inventory (STRAIN; [14]), an online assessment tool assessing self-reported lifetime stressor count, severity, timing, and duration of exposure, likewise found positive associations between greater lifetime stressor exposure and hair cortisol [18]. Furthermore, exploratory analyses from this study indicated that associations with stress exposure differed across life stages (i.e., cumulative stressor exposure during early life vs. adulthood). This work highlights the potential utility of cumulative stressor exposure measures in hair cortisol research to more thoroughly capture the complexity of individual stress experiences. To date, however, there is a distinct lack of studies combining indices of lifetime stressor exposure with an in-depth assessment of recent stressor exposure, as well as repeated, longitudinal measurement of hair cortisol. Likewise, hair cortisol research employing EMA-based assessments of cumulative daily life stress is scarce and findings are highly heterogeneous [19,20], thus highlighting the need for further investigation.

To address these gaps, we sought to clarify how different aspects of chronic stress exposure are related to hair cortisol across two studies, using both a cross-sectional (single time point; Study I) and mixed design (two time points; Study II). We applied the STRAIN across both studies to obtain high-quality self-report data on cumulative lifetime stressor exposure across multiple life domains. In Study II, we further incorporated detailed EMA-based stress assessments over three consecutive months as well as a three-month version of the STRAIN. Besides hair cortisol, we also examined the cortisol/dehydroepiandrosterone (DHEA) ratio as a complementary indicator of HPA axis activity. DHEA is released alongside cortisol as a result of HPA axis activation, but exerts counter-regulatory, anti-glucocorticoid, anti-inflammatory, and neuroprotective effects [[21], [22]]. Accordingly, the cortisol/DHEA ratio has been proposed to reflect the balance between catabolic and anabolic processes, with higher ratios indicating potential HPA axis dysregulation [23,24]. Against this background, we hypothesized that greater chronic stress exposure, assessed via different assessment tools, would be associated with higher hair cortisol levels and alterations in the cortisol/DHEA ratio. By combining comprehensive assessments of self-reported lifetime and momentary stress experience and examining hair cortisol alongside the cortisol/DHEA ratio across two methodologically aligned studies (one cross-sectional and one mixed design), the present work aims to provide a more differentiated picture of how perceived chronic stress is reflected in long-term endocrine activity.

## 2 Methods

### 2.1 Participants and procedure

**Fig. 1:**
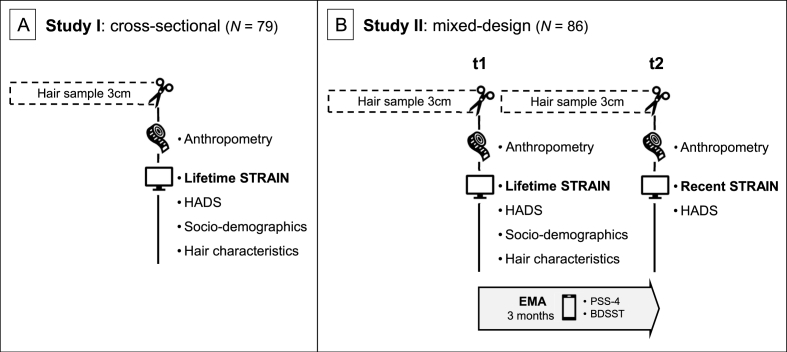
Overview of the research designs of Studies I and II. Participants attended either one laboratory session in the cross-sectional design of Study I (A) or two laboratory sessions, three months apart, in Study II (B). STRAIN = Stress and Adversity Inventory; HADS = Hospital Anxiety and Depression Scale; EMA = ecological momentary assessment; PSS-4 = 4-item Perceived Stress Scale; BDSST = Brief Daily Stressors Screening Tool.

*Overall research set-up (Studies I and II):* An overview of the study designs is provided in Fig. 1. The present research was part of a larger project designed to enable an in-depth assessment of life stress-related associations (as reported here) while also addressing specific methodological questions, including the effects of hair sample storage duration [25] and sampling location [26]. The methodological aims also motivated some specific design choices that are of relevance for the present research: In Study I, hair sampling was conducted across four balanced sampling waves, each separated by three months, with an identical procedure across sampling waves (see Ref. [25] for details; OSF preregistration: https://osf.io/4qpjx). For Study II, the methodological objectives were to investigate potential influences of scalp sampling region (posterior vertex versus occipital region) and hair sampling method (single hair sample vs. accumulation of multiple smaller samples) on hair-derived analytes. These objectives were investigated across two sampling waves: an initial wave (*n* = 52, [26]; OSF preregistration: https://osf.io/eu7vq) and a second wave approximately six months later (*n* = 36; methodological data unpublished; OSF preregistration: https://osf.io/pjhyu). Identical procedures were used across sampling waves, except for some additional hair sampling data being obtained in each wave (described below). Both study protocols were approved by the Ethics Committee of the University of Siegen and conducted in accordance with the Declaration of Helsinki. Prior to participation, all individuals provided written informed consent.

*Study I*. A total of 80 participants were recruited via flyers and digital resources of the University of Siegen (e.g., billboards, websites, email distribution lists). Participants had to be between 18 and 65 years of age and were required to have a minimum hair length of 3 cm at the back of their head. Exclusion criteria included severe or unusual hair loss, diagnosis of a mental disorder, current psychotherapeutic treatment, acute illness requiring medical evaluation, endocrinological disorders (e.g., Addison's disease, Cushing's syndrome), or use of any medication affecting mood, attention, or endocrine function (e.g., psychotropic substances, glucocorticoid-containing medication), as well as pregnancy (in female participants). Participants attended a single laboratory session during which they provided hair samples, anthropometric measures, stress-related and demographic information. Waist-to-hip ratio (WHR) was measured according to the recommendations of the World Health Organization [27]. Subsequently, three hair strands were sampled from the posterior vertex region (as defined by Ref. [26]). The laboratory session concluded with digital questionnaires assessing information on socio-demographics (e.g., sex, age), health (e.g., health condition, consumption of stimulants, physical activity), anxiety and depressive symptoms (Hospital Anxiety and Depression Scale; HADS), hair characteristics (i.e., natural hair color, hair coloring or bleaching within the last 3 months, regular heat exposure) and stressful life events (Stress and Adversity Inventory; STRAIN; see Section 2.2). As STRAIN data were lost for one individual due to technical issues, the final sample comprised 79 participants (66 female) with a mean (±SD) age of 24.67 (±6.43) years.

*Study II*. A total of 88 individuals were recruited for this study. Inclusion and exclusion criteria were identical to those of Study I, except for the additional exclusion of oral contraceptive use, which has recently been shown to potentially alter hair steroid concentrations [28]. Participants attended two laboratory sessions, separated by three months. Each session followed the procedure described in Study I, including WHR measurements and assessment of stress-related (Recent STRAIN; see Section 2.2) and demographic data, hair and health characteristics, as well as anxiety and depressive symptoms (HADS). Within the three months between both laboratory sessions, participants completed a stress-related ecological momentary assessment (EMA) via mobile app (see Section 2.2). Two participants had missing data at time point two, which led to a final sample of 86 participants (67 female) with a mean (±SD) age of 24.12 (±7.64) years.

### 2.2 Measures

#### 2.2.1 Cumulative lifetime stress exposure: Stress and Adversity Inventory (STRAIN)

Cumulative lifetime stressor exposure was assessed using the German version of the Stress and Adversity Inventory (STRAIN; [14,29]), a self-administered online assessment evaluating exposure to a wide variety of self-reported acute and chronic stressors. Participants were presented with 55 core stressor items across 12 life domains (e.g., education, work, housing, financial, marital/partner relationship). By using an adaptive branching logic with follow-up questions assessing stressor severity, frequency, exposure timing, and duration, the STRAIN enables investigators to obtain a highly detailed and time-sensitive stressor characterization that includes both stressor exposure and experience. The STRAIN is well-validated and has demonstrated strong test–retest reliability over two to four weeks, good concurrent, discriminant, and predictive validity across several psychological, clinical, and biological outcomes, including cortisol [14,18,29,30].

In Studies I and II, participants completed the Lifetime Adult STRAIN at their first session in the laboratory, which evaluates stressors from the prenatal period up to the interview. For statistical analysis, we used cumulative indices regarding the total stressor exposure severity for early-life and adulthood stress to capture distinct periods of lifetime stress exposure. In Study II, participants were also given a version of the Recent STRAIN at time point two. This version is identical to the Lifetime STRAIN in stressor coverage and implementation but limits the stressor period to the past three months.

#### 2.2.2 Ecological momentary assessment (EMA)

For the EMA in Study II, participants completed repeated stress-related questionnaires on their smartphones over three months. Two mobile applications were used for data collection: the app movisensXS (movisens GmbH, Karlsruhe, Germany) was implemented in the first recruitment wave. However, as this app only runs on Android devices and several participants experienced connectivity issues, the EMA application was switched to m-Path (KU Leuven, Belgium; [31]) for the second recruitment wave. Importantly, both applications employed identical questionnaires and sampling schedules, ensuring methodological consistency. The EMA protocol for each participant covered the three-month period between t1 and t2. Participants received push notifications every other day at randomized times between 10:00 a.m. and 9:00 p.m., with a response window of 60 min. An adapted, four-item version of the Perceived Stress Scale (PSS; [32]; adapted from Ref. [33]) was administered every three days and the Brief Daily Stressors Screening Tool (BDSST; [34]) was sent every week on Sundays.

The EMA-adapted version of the PSS includes four items from the original scale (“unable to control the important things in my life”, “felt nervous and stressed”, “could not cope with all the things that I had to do”, “difficulties were piling up so high that I could not overcome them”) and has demonstrated high within and between-person reliability and good criterion validity [33]. In this study, we used the items from the German adaptation [35], which were slightly rephrased to capture the current stress experience. Thus, participants were asked to indicate how they felt “*in this moment*” on a five-point Likert scale ranging from “*not at all*” to “*extremely*”.

The BDSST includes 10 items and assesses subjective experience of general daily stressors covering several life domains (i.e., social obligations, family responsibilities, health problems, financial restrictions, education/occupation, secondary employment, housing situation, and social conflicts). The BDSST has demonstrated acceptable internal consistency, good construct validity and high test–retest reliability over one month [34]. For the present study, the instructions were adapted to refer to the past week instead of the past 12 months. Participants were asked to indicate how strongly they had been affected by the stressors “*over the past week*” on a five-point Likert scale ranging from “*not at all*” to “*very much*”.

For EMA data to be included, participants had to meet predefined compliance criteria. Specifically, they had to respond to at least 50% of all EMA prompts, covering roughly two months, to avoid data being clustered only at the beginning or end of the study. As there are no established rules for EMA compliance or exclusion criteria [36] and response rates are known to decline over time in longitudinal EMA designs [37], the 50% criterion was chosen to retain an adequate sample size while ensuring sufficient temporal coverage to obtain reliable within-person estimates. For subsequent statistical analyses, a global EMA index was computed as the z-standardized sum of PSS-4 and BDSST scores.

### 2.3 Hair analysis

All hair samples were collected from the posterior vertex region (definition according to Ref. [26]). The first proximal three cm of each hair strand were segmented to reflect approximately three months of hair growth [38]. In Study I, the segmented samples were thoroughly mixed to minimize potential influences of regional variation in hormone concentration [26], resulting in a single homogenized sample per individual. For Study II, three hair samples were obtained from the posterior vertex, which were stored and analyzed separately and later averaged prior to analysis. For the additional methodological objectives, further samples were obtained from the occipital region (first wave) and from the posterior vertex using a different method (second wave), which, however, were not used for the present analyses. To minimize the risk of adverse effects on hair endocrine data due to prolonged storage periods [25], hair samples were analyzed within two weeks (Study I) or four months (Study II). Before analysis, samples were wrapped in aluminum foil and stored in a dark and dry environment at room temperature. All hair samples were analyzed at Dresden LABservice GmbH (Clemens Kirschbaum, PhD, Dresden, Germany). Endocrine analyses were conducted on 7.5 mg of hair per sample following a well-established liquid chromatography-tandem mass spectrometry (LC-MS/MS) protocol [39] to quantify steroid hormone concentrations of cortisol and dehydroepiandrosterone (DHEA).

### 2.4 Data analysis

Statistical analyses were conducted using R (version 4.3.1). Hormone and stress-related data were mostly right-skewed and transformed using natural log (ln) or square-root (sqrt) transformations to approximate a normal distribution, if necessary. Outliers exceeding ±1.5 times the interquartile range from the median were winsorized (i.e., replaced with non-extreme maxima/minima). As hair samples were analyzed across different laboratory batches, batch effects were statistically controlled by residualizing hormone values prior to all analyses. Findings remained robust in sensitivity analyses using non-residualized hormone values. All continuous predictors were mean-centered prior to inclusion in the models. For models including the STRAIN, sensitivity analyses were conducted excluding participants with zero stressor severity to account for clustering of values at zero, which did not alter the observed associations. Effect sizes are reported as R^2^ values.

*Study I.* To examine the association between cumulative lifetime stress exposure and hair analytes in a cross-sectional design, linear regression models were computed, each including a lifetime STRAIN subscale as predictor (*early-life* or *adulthood stressor exposure*). Besides simple unadjusted models, a fully adjusted model was computed including both predictors and controlling for anxiety/depressive symptoms (HADS), age, sex, waist-to-hip ratio (WHR) and hair treatment (hair coloring, bleaching, or regular heat exposure), as these factors have been associated with hair cortisol concentrations in prior work [8,40,41].

*Study II.* Associations between cumulative lifetime stressor exposure and hair analytes across t1 and t2 were examined using unadjusted linear mixed-effects models, including time as fixed effect and a random intercept for participants. As in Study I, additional adjusted models including HADS, age, sex, WHR and hair treatment as covariates were conducted. Degrees of freedom were estimated using Satterthwaite's approximation. Further, associations between recent stress and changes in hair analyte concentrations between t1 and t2 (Δ-scores, reflecting changes across the assessment interval) were examined using unadjusted linear regression models including (A) recent stressor exposure from the Recent STRAIN (preceding three months) and (B) a global EMA index. These analyses were complemented by adjusted models including all covariates mentioned above.

Sensitivity analyses were conducted for all unadjusted models. The available sample sizes provided sufficient power (80%) to detect small to medium effects for the linear regression models. For the linear mixed-effects models, Monte Carlo simulations likewise indicated adequate power (>80%) to detect effects of small to medium magnitude.

## 3 Results

### 3.1 Study I

Table 1A provides descriptive information on sample characteristics, hair analytes, and self-report data. Simple linear regression models showed no evidence of an association between STRAIN adulthood stressor exposure (*b* = −0.01, *SE* = 0.03, *t*(72) = −0.10, *p* = .918) or STRAIN early-life stressor exposure (*b* = 0.02, *SE* = 0.04, *t*(72) = 0.51, *p* = .615) and hair cortisol levels. Corresponding model-based associations are provided in the Supplement (Fig. S1). This pattern was also seen in the adjusted model controlling for anxiety/depressive symptoms, age, sex, WHR and hair treatment (*F*(7, 66) = 0.62, *p* = .740, *R*^*2*^ = 0.06, adjusted *R*^*2*^ = −0.04), with none of the predictors reaching significance (all *ps* > 0.21). Similar results were found for the cortisol/DHEA ratio: Adulthood (*b* = −0.01, *SE* = 0.06, *t*(64) = −0.10, *p* = .926) and early-life stressor exposure (*b* = −0.04, *SE* = 0.08, *t*(64) = −0.49, *p* = .625) were not significantly related to the cortisol/DHEA ratio. Likewise, none of the predictors in the fully adjusted model reached significance (*F*(7, 58) = 0.80, *p* = .590, *R*^*2*^ = 0.09, adjusted *R*^*2*^ = −0.02; all *ps* > 0.20). Full parameter estimates of the adjusted models can be found in the Supplement (see Table S1).

**Table 1:** Descriptive sample characteristics across Study I and II.

Variable	A) Study I (*N* = 79)		B) Study II (*N* = 86)			
			t1		t2	
	*M* (*SD*)/*n* (%)	Range	*M* (*SD*)/*n* (%)	Range	*M* (*SD*)/*n* (%)	Range
Age, *M* (*SD*)	24.67 (6.43)	18 - 64	24.12 (7.67)	18 - 59	-	-
Female, n (%)	66 (83.5%)	-	67 (77.9%)	-	-	-

Hair treatment, n (%)	33 (41.8%)	-	21 (24.4%)	-	29 (33.7%)	-
HADS, *M* (*SD*)	8.62 (5.35)	0 - 24	9.70 (10.00)	0 - 22	10.22 (6.20)	0 - 27
WHR, *M* (*SD*)	0.79 (0.07)	0.65 - 0.93	0.76 (0.08)	0.55 - 0.96	0.76 (0.07)	0.59 - 0.93
**STRAIN data**						
STRAIN - Adulthood, *M* (*SD*)	22.53 (14.86)	0 - 72	19.63 (15.55)	0 - 64	-	-
STRAIN - Early-Life, *M* (*SD*)	10.39 (7.84)	0 - 35	13.34 (11.55)	0 - 71	-	-
STRAIN - Recent, *M* (*SD*)	-	-	-	-	5.73 (6.81)	0 - 35
**EMA data** (*n* = 60)						
Received prompts, *M* (*SD*)	-	-	-	-	40.10 (4.45)	24 - 43
Response rate (%), *M* (*SD*)	-	-	-	-	87.8% (11.01)	54.76 - 100.00
PSS4, *M* (*SD*)	-	-	-	-	3.68 (2.34)	0.21 - 10.73
BDSST, *M* (*SD*)	-	-	-	-	7.14 (4.36)	0.62 - 21.60
EMA index (z-standardized), *M* (*SD*)	-	-	-	-	−0.01 (0.88)	−1.43 - 2.66
**Hair analytes**						
Cortisol (pg/mg), *M* (*SD*)	4.28 (2.40)	1.05 - 13.08	3.64 (2.81)	0.88 - 17.54	3.25 (3.44)	0.12 - 24.49
DHEA (pg/mg), *M* (*SD*)	14.16 (28.72)	1.08 - 196.78	17.49 (25.30)	0.25 - 165.96	13.14 (13.21)	1.18 - 71.01
Cortisol/DHEA ratio (pg/mg), *M* (*SD*)	0.78 (0.90)	0.02 - 4.31	0.48 (0.90)	0.02 - 6.88	0.37 (0.51)	0.03 - 3.80

*Note*. DHEA, dehydroepiandrosterone; HADS, Hospital Anxiety and Depression Scale; WHR, waist-to-hip ratio; STRAIN, Stress and Adversity Inventory; PSS, Perceived Stress Scale; BDSST, Brief Daily Stressors Screening Tool.

Sample sizes for analytes vary slightly as some values were non-detectable during laboratory analysis (cortisol: Study I *n* = 74, Study II *n* = 85; DHEA: Study I *n* = 71, Study II *n* = 66; cortisol/DHEA ratio: Study I *n* = 66, Study II *n* = 65).

**Table 2:** Study II: Standardized coefficients (β) from unadjusted and adjusted models.

Predictor	Model	Cortisol		Cortisol/DHEA ratio	
		β	*p*	β	*p*

STRAIN: Adulthood	Unadjusted	**0.22**	**0.028**	0.03	0.763
	Adjusted	**0.29**	**0.016**	0.01	0.979
STRAIN: Early-Life	Unadjusted	−0.04	0.501	0.09	0.404
	Adjusted	−0.09	0.403	0.11	0.335

		Δ Cortisol		Δ Cortisol/DHEA ratio	

STRAIN: Recent	Unadjusted	0.02	0.859	−0.14	0.273
	Adjusted	−0.01	0.911	**−0.28**	**0.053**
EMA Stress Index	Unadjusted	0.12	0.376	−0.14	0.361
	Adjusted	0.11	0.565	−0.05	0.817

*Note*. Standardized coefficients (β) reflect effect sizes derived from unadjusted and covariate-adjusted models predicting hair cortisol (ln, residualized) or the cortisol/DHEA ratio (ln, residualized). Δ values represent change scores from t1 to t2. Associations with p < .10 are highlighted in bold.

### 3.2 Study II

Descriptive information on sample characteristics, hair analytes, questionnaire and EMA data is provided in Table 1B. Standardized coefficients and p-values for the models are reported in Table 2. Full parameter estimates of adjusted models are shown in the Supplement (see Table S2).

#### 3.2.1 Cumulative lifetime stressor exposure

**Fig. 2:**
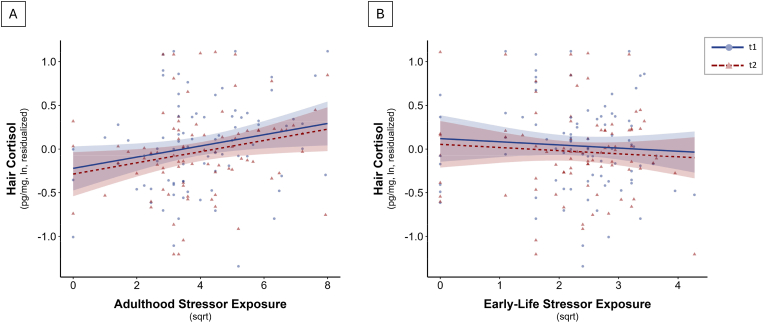
Study II: model-based associations (and 95% confidence intervals) between hair cortisol and stressor exposure in (A) adulthood or (B) early-life across time point one (t1) and time point two (t2). Points represent individual observations. Greater adulthood stressor exposure was associated with higher hair cortisol levels across both time points, whereas early-life stressor exposure showed no significant association.

Linear mixed-effects models revealed a significant positive association between STRAIN adulthood stressor exposure and hair cortisol (*b* = 0.06, *SE* = 0.03, *t*(83) = 2.24, *p* = .028), indicating that greater self-reported stressor exposure in adulthood was linked to higher hair cortisol levels. Including an interaction term between adulthood stressor exposure and time did not suggest that the association differed between time points (interaction: *b* = −0.01, *SE* = 0.03, *t*(83) = −0.44, *p* = .662). Early-life stressor exposure as assessed by the STRAIN showed no significant association with hair cortisol (*b* = −0.04, *SE* = 0.05, *t*(83) = −0.68, *p* = .501). Model-based associations are visualized in Fig. 2. The effect of adulthood stressor exposure remained significant in the fully adjusted model controlling for age, sex, WHR, HADS and hair treatment (*b* = 0.09, *SE* = 0.04, *t*(87.21) = 2.47, *p* = .016), while no other predictors reached significance (*R*^*2*^*m* = 0.07, *R*^*2*^*c* = 0.64; all *ps* > 0.23). By contrast, early-life (b = 0.08, SE = 0.10, t(63) = 0.84, p = .404) and adulthood stressor exposure (b = 0.02, SE = 0.05, t(63) = 0.30, p = .763) were unrelated to the cortisol/DHEA ratio. Again, this pattern of results remained unchanged in the fully adjusted model. Notably, time emerged as a significant predictor, both in the unadjusted and in the adjusted model (*b* = −0.28, *SE* = 0.09, *t*(64.99) = −3.21, *p* = .002), indicating that cortisol/DHEA ratios were significantly lower at t2 compared to t1. No other covariates were significant (*R*^*2*^*m* = 0.09, *R*^*2*^*c* = 0.66; all *ps* > 0.20).

#### 3.2.2 Recent stress and changes in hair analyte concentrations (Δ)

There were no significant associations between changes in hair cortisol (Δ) and recent self-reported stressor exposure as assessed by the STRAIN (*b* = 0.01, *SE* = 0.04, *t*(83) = 0.18, *p* = .859) or the EMA stress index (*b* = 0.06, *SE* = 0.07, *t*(57) = 0.89, *p* = .376). This pattern remained in the fully adjusted models (Recent STRAIN: *F*(6, 78) = 0.25, *p* = .957, *R*^*2*^ = 0.02, adjusted *R*^*2*^ = −0.06; EMA stress index: *F*(6, 52) = 0.36, *p* = .899, *R*^*2*^ = 0.04, adjusted *R*^*2*^ = −0.07), with none of the predictors reaching significance (all *ps* > 0.51). Similarly, for Δ cortisol/DHEA ratio, neither recent stressor exposure (*b* = −0.09, *SE* = 0.08, *t*(63) = −1.11, *p* = .273) nor the EMA stress index (*b* = 0.12, *SE* = 0.14, *t*(42) = 0.86, *p* = .393) were significantly associated. In the fully adjusted models (Recent STRAIN: *F*(6, 58) = 2.11, *p* = .065, *R*^*2*^ = 0.18, adjusted *R*^*2*^ = 0.09; EMA stress index: *F*(6, 37) = 0.81, *p* = .565, *R*^*2*^ = 0.12, adjusted *R*^*2*^ = −0.03), greater recent stressor exposure was marginally associated with a greater decrease in cortisol/DHEA ratio from t1 to t2 (*b* = −0.18, *SE* = 0.09, *t*(58) = −1.98, *p* = .053). Further, age was significantly associated with Δ cortisol/DHEA ratio in the recent stressor exposure model, indicating that older participants showed larger decreases across time points (*b* = −0.03, *SE* = 0.01, *t*(58) = −2.20, *p* = .032). A similar non-significant trend was observed in the EMA model (*b* = −0.03, *SE* = 0.02, *t*(37) = −1.79, *p* = .082). No other covariates were significant (all *ps* > 0.16).

## 4 Discussion

The present research employed detailed assessments of self-reported lifetime stressor exposure and EMA-based measures of daily stress experience to investigate associations with hair cortisol concentrations and the hair cortisol/DHEA ratio. Our most noteworthy finding is that in Study II, individuals who reported greater stressor severity in adulthood consistently exhibited elevated hair cortisol levels across both time points. This association was of moderate magnitude and remained robust across unadjusted and covariate-adjusted models. Surprisingly, a corresponding association between adult stressor exposure and hair cortisol was not seen in Study I. Moreover, hair cortisol was not related to self-reported early-life or recent stressor exposure. Likewise, no consistent associations were seen with the cortisol/DHEA ratio (included as an additional, alternative measure), although tentative evidence pointed towards a negative link between changes in cortisol/DHEA ratio across time and recent stressor exposure. These findings echo the heterogeneity and complexity of current evidence on the link between hair cortisol and chronic stress as discussed further below.

Our finding of a positive association between greater self-reported adult stressor exposure severity and hair cortisol levels across both time points in Study II is consistent with evidence from meta-analyses and systematic reviews indicating positive associations between different types of (ongoing) chronic stressors and hair cortisol in adults [8,17,42]. This work supports the idea that, under certain conditions, long-term cortisol output as measured by hair may be upregulated as a result of enduring cumulative stressor exposure. However, the absence of this association in Study I indicates that this link may not always be present and depends on contextual, methodological, or sample-specific characteristics, even across two methodologically aligned studies with comparable samples. Self-reported early-life stressor exposure, by contrast, was not associated with hair cortisol levels in either study. While null findings were reported by some prior studies [[43], [44]], other existing data showed either elevated or attenuated hair cortisol in adulthood following experiences of early adversity [17,18,40,45,46]. This includes a recent study by our group which also used STRAIN-based assessments in a healthy sample and found early-life stressor exposure to be a dominant determinant of hair cortisol [18]. Taken together, the heterogeneity of findings suggests that early-life stress exposure may be associated with divergent long-term trajectories of HPA axis adaptation. Regarding recent stressor exposure, we found no associations between recent stress indices and hair cortisol, regardless of whether stress exposure over the recent three months was assessed retrospectively via the STRAIN or aggregated by EMA. This contrasts with prior work reporting elevated hair cortisol levels in individuals reporting severe stressor exposure across a three-month period [16], but is consistent with research revealing no association between hair cortisol and recent stress assessed over periods ranging from three months to one year [[19], [43]]. Notably, the comparability across these studies is limited as assessment methods of recent stress differed substantially, ranging from single dichotomous items [16] to EMA approaches [19].

The observation that self-reported adulthood stressor exposure, but not recent stress, was associated with elevated hair cortisol is somewhat counterintuitive, given that hair cortisol is assumed to particularly capture hormone secretion over the recent period that corresponds to the respective hair growth phase [3,4]. One possible interpretation is that measures of cumulative adulthood stressor exposure may capture more severe and enduring stress experiences, some of which may still be ongoing and thus exert sustained effects on HPA axis functioning. This interpretation is consistent with integrative clinical and trauma-related models [[42], [47]], as well as with overall meta-analytical evidence [8] indicating dose- and time-dependent alterations in long-term cortisol secretion following stress exposure, including an initial upregulation in the context of severe or ongoing stress. Furthermore, episodes of severe stress may leave a lasting imprint on the neurobiological substrates of stress reactivity, thereby potentially inducing persistent changes in patterns of cortisol secretion and, thus, its relationship with hair cortisol. By contrast, recent stress measures may primarily reflect novel, transient stress experiences, which in healthy, non-clinical samples may mostly be of lower intensity. It is conceivable that such stressors may not exceed the threshold required to produce detectable changes in long-term cortisol output as reflected in hair samples. Consistent with this possibility, Karlén et al. [16] reported elevated hair cortisol concentrations over three months only in relation to the occurrence of *serious life events* (e.g., death of a close relative, serious illness, divorce). This suggests that severity and potential life-altering nature of stressors may be more critical determinants of hair cortisol than the overall amount of aggregated stress. Further, a longitudinal study showed that hair cortisol sharply increased with the onset of medical internship, declined as the stressor became familiar and rose again at the end of the internship [10]. Notably, hair cortisol changes in this study were not predicted by perceived stress or workload but appeared to be driven by contextual characteristics such as novelty, anticipation, and social-evaluative threat. Consequently, it might not be enough to distinguish between recent and lifetime stressors, but rather whether stress exposure reflects more severe, persistent strains as opposed to fluctuating, episodic experiences. Indeed, the descriptive data of the recent STRAIN in our research show low average stressor severity and a strongly right-skewed distribution. This concurs with the possibility that stressors captured during the past three months were, for most participants, insufficiently severe or persistent to induce detectable changes in cumulative cortisol output.

Beyond hair cortisol, we also examined the cortisol/DHEA ratio as a potential marker of HPA axis balance and stress-related dysregulation [23,24]. Overall, we did not observe robust associations between the cortisol/DHEA ratio and either recent or lifetime stress indices. In Study II, however, a non-significant trend for a negative association between changes in the cortisol/DHEA ratio across time and recent stressor exposure emerged. This may tentatively point toward stress-related alterations in the relative balance of cortisol and DHEA. Descriptive data indicated a decline in both cortisol and DHEA concentrations from time point one to time point two, which was more pronounced in DHEA. Accordingly, the observed changes in the cortisol/DHEA ratio appear to reflect differential trajectories of the two hormones over time and may be more pronounced under conditions of higher recent stress, although interpretations must remain cautious. The absence of consistent associations between the cortisol/DHEA ratio and stress exposure is consistent with the heterogeneous empirical evidence to date. A recent systematic review and meta-analysis concluded that available data are limited and do not support a reliable association with chronic stress [48]. Possibly, alterations in cortisol/DHEA balance may be more reliably detectable in clinical samples characterized by pronounced dysregulation [49]. Our data thus add to the notion that the utility of the cortisol/DHEA ratio as a marker of chronic stress, particularly in non-clinical samples, remains insufficiently established and warrants further systematic investigation.

Overall, despite providing partial evidence for elevated hair cortisol in individuals reporting higher adulthood stressor exposure, the present findings also highlight the complexity of potential links between hair cortisol and self-reported chronic stress and that our current understanding of this is still rather limited. Future research may benefit from integrating hair cortisol assessments within a broader, multi-method framework of stress-related biomarkers. Using longitudinal research designs and combining hair-based measures with complementary approaches targeting different aspects of HPA axis activity (e.g., repeated salivary cortisol sampling, dexamethasone suppression tests, assessments of glucocorticoid receptor number and sensitivity) may help disentangle stress-related changes from stable individual differences in HPA axis functioning [47].

In addition, it should be acknowledged that although we used more elaborate stress assessment methods (i.e., the STRAIN and EMA-based measures), these approaches still depend on participants' self-reports, which can be inaccurate or biased, particularly for stressors occurring in the distant past. In this context, it is noteworthy that prior research reporting strong associations between hair cortisol and early-life stress used methodologies that combined self-report data with objective indicators of stressor exposure (e.g., child protection service records of early maltreatment; [45]). At the same time, although adults’ retrospective reports of major adverse childhood experiences may be subject to bias, particularly with respect to minor details and more ambiguous events, this bias does not appear to be substantial enough to invalidate retrospective reports [50]. Therefore, while the reliance on self-report data may have attenuated associations in the present research, it is unlikely to fully account for the overall pattern of findings.

Some further limitations should also be considered. Although the sample sizes were adequate to detect linear associations, they may have been too limited to detect more complex patterns, including non-linear or subgroup-specific effects. In addition, the relatively selective eligibility criteria may constrain the extent to which these findings generalize beyond the present samples. Besides conceptual considerations, methodological factors are likely to further contribute to heterogeneous findings. Variability in sampling region [26], storage duration [25], interindividual hair growth rate [51], preprocessing procedures and selection of covariates may all influence hair cortisol results and limit comparability across studies, thus emphasizing the need for greater methodological standardization and transparency in future research.

In conclusion, our findings provide only partial support for an association between self-reported chronic stress and hair cortisol, while at the same time underscoring the complexity and context-dependency of this relationship. Moving beyond the notion of a simple and uniform biomarker-stress relationship, hair cortisol may be most informative under specific conceptual and methodological conditions. Clarifying these conditions will be a key challenge for future psychoneuroendocrine research.

## CRediT authorship contribution statement

**Katharina Huthsteiner:** Writing – original draft, Visualization, Validation, Project administration, Methodology, Investigation, Formal analysis, Data curation, Conceptualization. **Johannes B. Finke:** Writing – review & editing, Supervision, Methodology, Formal analysis. **Jari Planert:** Writing – review & editing, Investigation. **George M. Slavich:** Writing – review & editing, Software, Methodology. **Tim Klucken:** Writing – review & editing, Supervision, Resources. **Tobias Stalder:** Writing – review & editing, Supervision, Resources, Project administration, Methodology, Funding acquisition, Conceptualization.

## Funding supporting the writing of the article

The current project received funding from the German Research Foundation (DFG; STA 1213/11-1; project number: 503208201). G. M. Slavich was supported by grant #OPR21101 from the California Governor's Office of Planning and Research/California Initiative to Advance Precision Medicine. The findings and conclusions in this article are those of the authors and do not necessarily represent the views or opinions of these organizations, which had no role in designing or planning this study; in collecting, analyzing, or interpreting the data; in writing the article; or in deciding to submit this article for publication.

## Declaration of competing interest

The authors have no conflicts of interest to declare.

## References

[1] Chrousos G.P. (2009). Stress and disorders of the stress system. Nat. Rev. Endocrinol..

[2] Kirschbaum C., Tietze A., Skoluda N., Dettenborn L. (2009). Hair as a retrospective calendar of cortisol production-increased cortisol incorporation into hair in the third trimester of pregnancy. Psychoneuroendocrinology.

[3] Russell E., Koren G., Rieder M., van Uum S. (2012). Hair cortisol as a biological marker of chronic stress: current status, future directions and unanswered questions. Psychoneuroendocrinology.

[4] Stalder T., Kirschbaum C. (2012). Analysis of cortisol in hair--state of the art and future directions. Brain Behav. Immun..

[5] Stalder T., Steudte S., Miller R., Skoluda N., Dettenborn L., Kirschbaum C. (2012). Intraindividual stability of hair cortisol concentrations. Psychoneuroendocrinology.

[6] Short S.J., Stalder T., Marceau K., Entringer S., Moog N.K., Shirtcliff E.A. (2016). Correspondence between hair cortisol concentrations and 30-day integrated daily salivary and weekly urinary cortisol measures. Psychoneuroendocrinology.

[7] Manenschijn L., Koper J.W., Lamberts S.W.J., van Rossum E.F.C. (2011). Evaluation of a method to measure long term cortisol levels. Steroids.

[8] Stalder T., Steudte-Schmiedgen S., Alexander N., Klucken T., Vater A., Wichmann S. (2017). Stress-related and basic determinants of hair cortisol in humans: a meta-analysis. Psychoneuroendocrinology.

[9] Staufenbiel S.M., Penninx B.W.J.H., Spijker A.T., Elzinga B.M., van Rossum E.F.C. (2013). Hair cortisol, stress exposure, and mental health in humans: a systematic review. Psychoneuroendocrinology.

[10] Mayer S.E., Lopez-Duran N.L., Sen S., Abelson J.L. (2018). Chronic stress, hair cortisol and depression: a prospective and longitudinal study of medical internship. Psychoneuroendocrinology.

[11] Planert J., Klucken T., Finke J.B., Paulus P.C., Fischer J.E., Gao W. (2023). Associations between hair cortisol and subjective stress measures in a large occupational sample. Psychoneuroendocrinology.

[12] Kim S.J., Karayeva E., Negrete M., Bendinskas K., Winn R.A., Matthews A.K. (2025). Neighborhood violence, hair cortisol, and perceived stress among Black men living in a large urban city. Psychoneuroendocrinology.

[13] Shields G.S., Slavich G.M. (2017). Lifetime stress exposure and health: a review of contemporary assessment methods and biological mechanisms. Soc. Personal. Psychol. Compass.

[14] Slavich G.M., Shields G.S. (2018). Assessing lifetime stress exposure using the stress and adversity inventory for adults (Adult STRAIN): an overview and initial validation. Psychosom. Med..

[15] Shiffman S., Stone A.A., Hufford M.R. (2008). Ecological momentary assessment. Annu. Rev. Clin. Psychol..

[16] Karlén J., Ludvigsson J., Frostell A., Theodorsson E., Faresjö T. (2011). Cortisol in hair measured in young adults - a biomarker of major life stressors?. BMC Clin. Pathol..

[17] Khoury J.E., Bosquet Enlow M., Plamondon A., Lyons-Ruth K. (2019). The association between adversity and hair cortisol levels in humans: a meta-analysis. Psychoneuroendocrinology.

[18] Planert J., Stalder T., Huthsteiner K., Slavich G.M., Klucken T., Finke J.B. (2026). Linking hair cortisol and life stress: the role of stress reactivity and habituation. Psychoneuroendocrinology.

[19] Gidlow C.J., Randall J., Gillman J., Silk S., Jones M.V. (2016). Hair cortisol and self-reported stress in healthy, working adults. Psychoneuroendocrinology.

[20] Kozusznik M.W., Euwema M.C. (2020). Start-up conflict and hair cortisol. Psychoneuroendocrinology.

[21] Dutheil F., Saint Vincent S de, Pereira B., Schmidt J., Moustafa F., Charkhabi M. (2021). DHEA as a biomarker of stress: a systematic review and meta-analysis. Front. Psychiatr..

[22] Mouthaan J., Sijbrandij M., Luitse J.S.K., Goslings J.C., Gersons B.P.R., Olff M. (2014). The role of acute cortisol and DHEAS in predicting acute and chronic PTSD symptoms. Psychoneuroendocrinology.

[23] Schulz A., Bellingrath S., Lutz A., Kumsta R., Vögele C. (2025). Acute and chronic stress effects on cardiac interoceptive accuracy and heartbeat-evoked potentials in chronically-stressed schoolteachers. Psychoneuroendocrinology.

[24] Schultchen D., Bayer J., Kühnel J., Melchers K.G., Pollatos O. (2019). Interoceptive accuracy is related to long-term stress via self-regulation. Psychophysiology.

[25] Huthsteiner K., Finke J.B., Peters E.M.J., Klucken T., Stalder T. (2025). Hair cortisol and other hair analytes decline with storage over a one-year period: a systematic, within-subject investigation. Psychoneuroendocrinology.

[26] Huthsteiner K., Finke J.B., Peters E.M.J., Kleinke K., Klucken T., Stalder T. (2025). What is the best sampling region for endocrine hair analysis? A comparison between the posterior vertex and occipital region and recommendation for standardization. Psychoneuroendocrinology.

[27] WHO (2011).

[28] Carrillo Vázquez M., Johnson-Ferguson L., Zimmermann J., Baumgartner M.R., Binz T.M., Beuschlein F. (2022). Associations of different hormonal contraceptive methods with hair concentrations of cortisol, cortisone, and testosterone in young women. Compr. Psychoneuroendocrinol..

[29] Sturmbauer S.C., Shields G.S., Hetzel E.-L., Rohleder N., Slavich G.M. (2019). The stress and adversity inventory for adults (Adult STRAIN) in German: an overview and initial validation. PLoS One.

[30] Lam J.C.W., Shields G.S., Trainor B.C., Slavich G.M., Yonelinas A.P. (2019). Greater lifetime stress exposure predicts blunted cortisol but heightened DHEA responses to acute stress. Stress Health.

[31] Mestdagh M., Verdonck S., Piot M., Niemeijer K., Kilani G., Tuerlinckx F. (2023). m-Path: an easy-to-use and highly tailorable platform for ecological momentary assessment and intervention in behavioral research and clinical practice. Front. Digit. Health.

[32] Cohen S., Kamarck T., Mermelstein R. (1983). A global measure of perceived stress. J. Health Soc. Behav..

[33] Murray A.L., Xiao Z., Zhu X., Speyer L.G., Yang Y., Brown R.H. (2023). Psychometric evaluation of an adapted version of the perceived stress scale for ecological momentary assessment research. Stress Health.

[34] Scholten S., Lavallee K., Velten J., Zhang X.-C., Margraf J. (2020). The brief daily stressors screening tool: an introduction and evaluation. Stress Health.

[35] Schneider E.E., Schönfelder S., Domke-Wolf M., Wessa M. (2020). Measuring stress in clinical and nonclinical subjects using a German adaptation of the perceived stress scale. Int. J. Clin. Health Psychol..

[36] Stone A.A., Shiffman S. (2002). Capturing momentary, self-report data: a proposal for reporting guidelines. Ann. Behav. Med..

[37] Tonkin S., Gass J., Wray J., Maguin E., Mahoney M., Colder C. (2023). Evaluating declines in compliance with ecological momentary assessment in longitudinal health behavior research: analyses from a clinical trial. J. Med. Internet Res..

[38] Wennig R. (2000). Potential problems with the interpretation of hair analysis results. Forensic Sci. Int..

[39] Gao W., Stalder T., Foley P., Rauh M., Deng H., Kirschbaum C. (2013). Quantitative analysis of steroid hormones in human hair using a column-switching LC-APCI-MS/MS assay. J. Chromatogr., B: Anal. Technol. Biomed. Life Sci..

[40] Abell J.G., Stalder T., Ferrie J.E., Shipley M.J., Kirschbaum C., Kivimäki M. (2016). Assessing cortisol from hair samples in a large observational cohort: the Whitehall II study. Psychoneuroendocrinology.

[41] Dettenborn L., Muhtz C., Skoluda N., Stalder T., Steudte S., Hinkelmann K. (2012). Introducing a novel method to assess cumulative steroid concentrations: increased hair cortisol concentrations over 6 months in medicated patients with depression. Stress.

[42] Schindler-Gmelch L., Capito K., Steudte-Schmiedgen S., Kirschbaum C., Berking M. (2024). Hair cortisol research in posttraumatic stress disorder - 10 years of insights and open questions. A systematic review. Curr. Neuropharmacol..

[43] Oresta S., Vinkers C.H., van Rossum E.F.C., Penninx B.W.J.H., Nawijn L. (2021). How childhood trauma and recent adverse events are related to hair cortisol levels in a large adult cohort. Psychoneuroendocrinology.

[44] Hummel K.V., Schellong J., Trautmann S., Kummer S., Hürrig S., Klose M. (2021). The predictive role of hair cortisol concentrations for treatment outcome in PTSD inpatients. Psychoneuroendocrinology.

[45] White L.O., Ising M., Klitzing K von, Sierau S., Michel A., Klein A.M. (2017). Reduced hair cortisol after maltreatment mediates externalizing symptoms in middle childhood and adolescence. JCPP (J. Child Psychol. Psychiatry).

[46] Marsland A.L., Jones E., Reed R.G., Walsh C.P., Natale B.N., Lindsay E.K. (2024). Childhood trauma and hair cortisol response over the year following onset of a chronic life event stressor. Psychoneuroendocrinology.

[47] Steudte-Schmiedgen S., Kirschbaum C., Alexander N., Stalder T. (2016). An integrative model linking traumatization, cortisol dysregulation and posttraumatic stress disorder: insight from recent hair cortisol findings. Neurosci. Biobehav. Rev..

[48] Huchegowda R., Kulkarni R.R., Bijjal S., Nagesh P.N., Philip M., Harbishettar V. (2025). Association between hair cortisol, dehydroepiandrosterone and perceived stress in chronic stress-related conditions: a systematic review and meta-analysis. Indian J. Psychol. Med..

[49] Pividori I., Peric T., Comin A., Cotticelli A., Corazzin M., Prandi A. (2024). Hair Cortisol/DHEA-S ratios in healthcare workers and their patients during the COVID-19 pandemic: a case study. Life.

[50] Hardt J., Rutter M. (2004). Validity of adult retrospective reports of adverse childhood experiences: review of the evidence. JCPP (J. Child Psychol. Psychiatry).

[51] LeBeau M.A., Montgomery M.A., Brewer J.D. (2011). The role of variations in growth rate and sample collection on interpreting results of segmental analyses of hair. Forensic Sci. Int..

