# Supplementary material for: From lifetime stressor exposure to daily stress experience: Associations with hair cortisol
Source: Compr Psychoneuroendocrinol. 2026 Apr 8;26:100348. doi: 10.1016/j.cpnec.2026.100348 (PMC13091833; doi:10.1016/j.cpnec.2026.100348)
Supplement: Multimedia component 1 [file mmc1.docx]

| **Table S1.**  Study I: Model parameters for lifetime stressor exposure on hair cortisol and cortisol/DHEA ratio (Multiple linear regression) | | | | | | | | | | | | |
| --- | --- | --- | --- | --- | --- | --- | --- | --- | --- | --- | --- | --- |
|  |  | **Cortisol** | | | | |  | **Cortisol/DHEA ratio** | | | | |
| Predictor |  | β | *b* | *SE* | *t* | *p* |  | β | *b* | *SE* | *t* | *p* |
|  |  |  |  |  |  |  |  |  |  |  |  |  |
| (Intercept) |  | - | -0.03 | 0.16 | -0.17 | .864 |  | - | -0.41 | 0.31 | -1.33 | .189 |
| STRAIN: Adulthood |  | -0.06 | -0.02 | 0.07 | -0.40 | .697 |  | 0.06 | 0.03 | 0.13 | 0.42 | .676 |
| STRAIN: Early-life |  | 0.10 | 0.04 | 0.07 | 0.77 | .445 |  | 0.02 | 0.01 | 0.12 | 0.16 | .876 |
| Anxiety/Depression |  | -0.04 | -0.02 | 0.07 | -0.29 | .772 |  | -0.15 | -0.13 | 0.12 | -1.00 | .320 |
| Age |  | 0.14 | 0.01 | 0.09 | 1.04 | .300 |  | -0.11 | -0.02 | 0.16 | -0.76 | .449 |
| Sex |  | 0.09 | 0.12 | 0.18 | 0.65 | .516 |  | 0.18 | 0.44 | 0.34 | 1.28 | .204 |
| WHR |  | 0.06 | 0.46 | 0.06 | 0.49 | .626 |  | -0.11 | -1.55 | 0.12 | -0.86 | .391 |
| Hair treatment |  | -0.16 | -0.17 | 0.13 | -1.28 | .207 |  | 0.04 | 0.06 | 0.24 | 0.27 | .787 |
|  |  |  |  |  |  |  |  |  |  |  |  |  |
| *Note*. Standardized (β) and unstandardized coefficients (*b*), standard error (*SE*) derived from covariate-adjusted models predicting hair hormone concentrations (ln, residualized).  DHEA, dehydroepiandrosterone; STRAIN, Stress and Adversity Inventory; WHR, waist-to-hip ratio. | | | | | | | | | | | | |

| **Table S2.**  Study II: Model parameters for lifetime and recent stressor exposure on hair cortisol and cortisol/DHEA ratio | | | | | | | | | | | | | | | |
| --- | --- | --- | --- | --- | --- | --- | --- | --- | --- | --- | --- | --- | --- | --- | --- |
|  |  | **Cortisol** | | | | | |  | **Cortisol/DHEA ratio** | | | | | |  |
| Predictor |  | β | *b* | *SE* | *t* | *df* | ***p*** |  | β | *b* | *SE* | *t* | *df* | ***p*** |  |
|  |  |  |  |  |  |  |  |  |  |  |  |  |  |  |  |
| **A) Lifetime stressor exposure** (LME) | | | | | | | | | | | | | | | |
|  |  |  |  |  |  |  |  |  |  |  |  |  |  |  |  |
| (Intercept) |  | - | -0.04 | 0.12 | -0.33 | 99.02 | .739 |  | - | -0.12 | 0.21 | -0.57 | 77.01 | .573 |  |
| Time |  | -0.12 | -0.06 | 0.05 | -1.21 | 83.83 | .229 |  | -0.35 | -0.28 | 0.09 | -3.21 | 64.99 | .002** |  |
| STRAIN: Adulthood |  | 0.29 | 0.09 | 0.03 | 2.47 | 87.21 | .016* |  | 0.01 | 0.01 | 0.06 | 0.03 | 68.08 | .979 |  |
| STRAIN: Early-life |  | -0.09 | -0.05 | 0.06 | -0.84 | 81.59 | .403 |  | 0.11 | 0.10 | 0.10 | 0.97 | 61.47 | .335 |  |
| Anxiety/Depression |  | -0.07 | -0.04 | 0.05 | -0.77 | 160.50 | .446 |  | -0.01 | -0.01 | 0.09 | -0.11 | 120.26 | .910 |  |
| Age |  | -0.11 | -0.01 | 0.01 | -0.92 | 95.02 | .362 |  | 0.10 | 0.01 | 0.01 | 0.74 | 73.85 | .463 |  |
| Sex |  | 0.22 | 0.12 | 0.14 | 0.80 | 102.72 | .425 |  | 0.36 | 0.29 | 0.25 | 1.17 | 80.92 | .245 |  |
| WHR |  | 0.08 | 0.53 | 0.77 | 0.69 | 160.97 | .489 |  | -0.04 | -0.38 | 1.37 | -0.28 | 120.95 | .781 |  |
| Hair treatment |  | -0.11 | -0.05 | 0.08 | -0.67 | 139.48 | .505 |  | 0.23 | 0.18 | 0.14 | 1.30 | 102.16 | .198 |  |
|  |  |  |  |  |  |  |  |  |  |  |  |  |  |  |  |
|  | | | | | | | | | | | | | | | |
| **B) Recent stressor exposure** (Δ hair cortisol/DHEA ratio; MLR) | | | | | | | | | | | | | | | |
|  |  |  |  |  |  |  |  |  |  |  |  |  |  |  |  |
| *Recent STRAIN* | | | | | | | | | | | | | | | |
|  |  |  |  |  |  |  |  |  |  |  |  |  |  |  |  |
| (Intercept) |  | - | -0.11 | 0.13 | -0.88 | 78 | .381 |  | - | -0.53 | 0.20 | -2.63 | 58 | .011* |  |
| STRAIN: Recent |  | -0.01 | -0.01 | 0.05 | -0.11 | 78 | .911 |  | -0.28 | -0.18 | 0.10 | -1.98 | 58 | .053° |  |
| Anxiety/Depression |  | 0.04 | 0.02 | 0.06 | 0.33 | 78 | .746 |  | 0.20 | 0.14 | 0.10 | 1.41 | 58 | .163 |  |
| Age |  | -0.10 | -0.01 | 0.01 | -0.66 | 78 | .511 |  | -0.34 | -0.03 | 0.01 | -2.20 | 58 | .032* |  |
| Sex |  | 0.05 | 0.05 | 0.16 | 0.34 | 78 | .735 |  | 0.18 | 0.29 | 0.26 | 1.12 | 58 | .268 |  |
| WHR |  | -0.01 | -0.02 | 1.01 | -0.02 | 78 | .983 |  | 0.13 | 1.21 | 1.61 | 0.75 | 58 | .455 |  |
| Hair treatment |  | 0.01 | 0.01 | 0.12 | 0.10 | 78 | .923 |  | 0.05 | 0.08 | 0.20 | 0.39 | 58 | .700 |  |
|  |  |  |  |  |  |  |  |  |  |  |  |  |  |  |  |
| *Ecological momentary assessment (EMA)* | | | | | | | | | | | | | | | |
|  |  |  |  |  |  |  |  |  |  |  |  |  |  |  |  |
| (Intercept) |  | - | -0.14 | 0.15 | -0.94 | 53 | .352 |  | - | -0.34 | 0.28 | -1.24 | 53 | .224 |  |
| EMA Stress Index |  | 0.11 | 0.05 | 0.09 | 0.58 | 53 | .565 |  | -0.05 | -0.05 | 0.22 | -0.23 | 53 | .817 |  |
| Anxiety/ Depression |  | 0.01 | < 0.01 | 0.08 | 0.01 | 53 | 1.00 |  | 0.10 | 0.07 | 0.17 | 0.44 | 53 | .664 |  |
| Age |  | -0.12 | -0.01 | 0.01 | -0.67 | 53 | .508 |  | -0.37 | -0.03 | 0.02 | -1.79 | 53 | .082° |  |
| Sex |  | 0.11 | 0.12 | 0.19 | 0.64 | 53 | .527 |  | 0.01 | 0.01 | 0.35 | 0.04 | 53 | .973 |  |
| WHR |  | 0.02 | 0.13 | 1.14 | 0.11 | 53 | .912 |  | 0.12 | 1.18 | 2.07 | 0.57 | 53 | .572 |  |
| Hair treatment |  | -0.07 | -0.06 | 0.14 | -0.43 | 53 | .669 |  | 0.04 | 0.07 | 0.27 | 0.25 | 53 | .802 |  |
|  |  |  |  |  |  |  |  |  |  |  |  |  |  |  |  |
| *Notes*. Standardized (β) and unstandardized coefficients (*b*), standard error (*SE*) derived from covariate-adjusted A) LME, Linear mixed effects models (over two time points) and B) MLR, multiple linear regression models. Δ, changes in hair cortisol/DHEA ratio between two time points. Degrees of freedom for LME were estimated using Satterthwaite’s approximation.  STRAIN, Stress and Adversity Inventory; EMA Stress Index, sum of PSS-4 and BDSST scores (z-standardized). p < .001**, p < .05*, p < .10°. | | | | | | | | | | | | | | | |
