# Supplementary material for: From lifetime stressor exposure to daily stress experience: Associations with hair cortisol
Source: Compr Psychoneuroendocrinol. 2026 Apr 8;26:100348. doi: 10.1016/j.cpnec.2026.100348 (PMC13091833; doi:10.1016/j.cpnec.2026.100348)
Supplement: Multimedia component 2 [file mmc2.pdf]

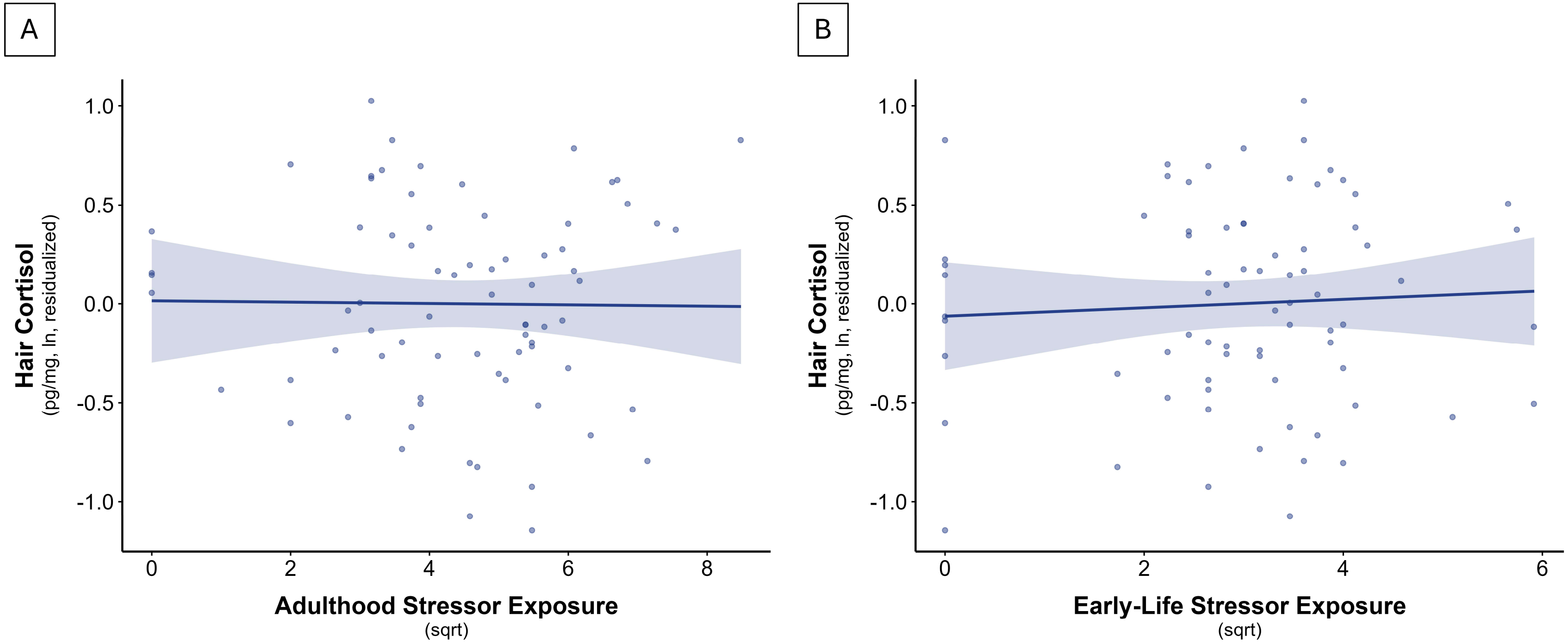

Figure S1. Study I: model-based associations (and 95% confidence intervals) between hair cortisol and stressor exposure in (A) adulthood or (B) early-life. Points represent individual observations.
